# Primer-Dependent Insights into Rumen Microbiota and Methanogen Shifts Induced by Orange Peel Secondary Feed in Dairy Sheep

**DOI:** 10.3390/ani15203041

**Published:** 2025-10-20

**Authors:** Maria-Anastasia Karatzia, Zoitsa Basdagianni, Sofia-Afroditi Termatzidou, Basiliki Kotsampasi, Eleni Kasapidou, Sofia Mai, Elli-Maria Barampouti, Maria V. Alvanou, Dimitrios Loukovitis

**Affiliations:** 1 Research Institute of Animal Science, Directorate General of Agricultural Research, Hellenic Agricultural Organization—DIMITRA, 58100 Giannitsa, Greece; termatzidou@heliadesvet.com (S.-A.T.); vkotsampasi@elgo.gr (B.K.); 2 Department of Animal Production, School of Agriculture, Aristotle University of Thessaloniki, 54124 Thessaloniki, Greece; basdagianni@agro.auth.gr (Z.B.); mariaalvanou7@gmail.com (M.V.A.); 3 Department of Agriculture, University of Western Macedonia, Terma Kontopoulou, 53100 Florina, Greece; ekasapidou@uowm.gr; 4 Unit of Environmental Science & Technology, School of Chemical Engineering, National Technical University of Athens, 9 Iroon Polytechniou Str., Zographou Campus, 15780 Athens, Greece; mai@central.ntua.gr (S.M.); belli@central.ntua.gr (E.-M.B.); 5 Department of Fisheries and Aquaculture, School of Agricultural Sciences, University of Patras, 30200 Messolonghi, Greece; dloukovi@upatras.gr

**Keywords:** microbial community profiling, archaeal diversity, citrus by-products, dairy ewes, methane

## Abstract

**Simple Summary:**

Dairy sheep farming is an integral part of the food production chain, but it faces challenges related to high feed costs and environmental impact, especially the release of methane, a strong greenhouse gas produced during digestion. In this study, we tested whether orange peel waste from the juice industry, either used as is or after processing, could be added to sheep diets to improve sustainability and reduce emissions. Over a period of 84 days, we found that including orange peel waste changed the community of microbes in the animals’ rumen in ways that may benefit both them and the environment. Animals receiving orange peel waste did not show an increase in bacteria linked to poor digestive health, as observed in the control group, and the processed orange peel reduced the presence of methane-producing microbes. These results suggest that orange peel waste, normally discarded, can be transformed into a valuable feed ingredient that reduces waste and could help limit greenhouse gas emissions.

**Abstract:**

Ruminant livestock production faces rising challenges related to feed costs, sustainability, and methane (CH_4_) emissions, with the rumen microbiome playing a central role. This study evaluated the effects of processed and unprocessed orange peel waste, valorized as secondary feedstuff, on rumen microbial composition and methanogen abundance in dairy sheep while assessing primer-dependent biases in microbial detection. Eighteen mid-lactation Chios ewes were assigned to three isonitrogenous and isoenergetic diets: control, 11% processed orange peel, and 11% unprocessed orange peel, over an 84-day trial. Rumen samples collected on days 0 and 84 were analyzed using Oxford Nanopore sequencing with full-length 16S (V1–V9) and prokaryotic (V3–V4) primers. Firmicutes (39.5–58.0%) and Bacteroidota (20.0–37.4%) predominated across diets, while Methanobacteria (6.9–8.8%) were detected exclusively with the prokaryotic primer. Orange peel inclusion attenuated the rise of Proteobacteria in controls and stabilized Prevotella populations. Notably, the processed orange peel diet reduced Methanobacteria abundance by 19.3% (*p* < 0.05) after 84 days, suggesting enhanced antimethanogenic effects. These results highlight both the methodological relevance of primer selection and the potential of citrus by-products as sustainable feed ingredients that promote rumen microbial stability and contribute to methane mitigation in dairy sheep production.

## 1. Introduction

Ruminant livestock plays a pivotal role in global food systems by supplying high-quality animal protein, contributing to food security, and supporting rural economies [1,2]. However, the digestive processes within the rumen generate significant amounts of methane (CH_4_), a highly impactful greenhouse gas possessing a global warming potential nearly 28 times greater than that of carbon dioxide over a 100-year timeframe [3,4]. Enteric methane emissions represent not only a major contributor to anthropogenic greenhouse gases, amounting to over 86 million tonnes annually, with 9.5 million tonnes attributed to sheep alone, but also a considerable loss of feed energy, up to 12% of gross intake [4,5,6]. Methane production diverts metabolic hydrogen away from more energy-efficient pathways, reducing overall energy utilization [7,8], although there are still controversies around the relative contributions of alternative hydrogen sinks such as acetogenesis and propionate production, and their potential to compete with methanogens [9,10,11]. Methanogenesis in ruminants is carried out primarily by archaea, especially those of the genus *Methanobrevibacter*, which utilize hydrogen and carbon dioxide as substrates, These microorganisms maintain the hydrogen balance in the rumen but also constitute a major target for greenhouse gas mitigation strategies [12,13]. As such, recent efforts have focused on rumen microbiome modulation via nutritional strategies to reduce CH_4_ production while maintaining or enhancing fermentation efficiency, animal health, and productivity [14,15]. A multitude of studies have demonstrated modest (~3%) to substantial (~80%) methane reductions, with feed additives such as 3-NOP, red seaweed, and lipids showing the highest efficacy [16,17,18]. Plant secondary metabolites such as tannins and saponins consistently reduced methane by 10–30% in multiple studies in sheep [19,20], while forage-based strategies including legumes and brassicas achieved reductions typically between 15–37%, with effectiveness influenced by inclusion levels and forage quality [21,22,23]. Some lipid supplements in sheep rations, especially medium-chain fatty acids and coconut oil, exhibited variable methane suppression depending on diet composition [24,25,26].

One promising nutritional strategy for rumen microbiome modulation involves the inclusion of agro-industrial by-products, particularly citrus-derived residues, in ruminant diets. The citrus processing industry, especially orange juice manufacturing, generates large volumes of by-products such as peels, pulp, and seeds, with roughly 50–60% of the fruit mass left as waste post-juice extraction [27,28]. In the European Union alone, orange waste is estimated at 2.5 million tonnes annually [29]. Traditionally considered an environmental burden, these residues offer a valuable opportunity to align circular economy principles with ruminant nutrition. Orange peels are rich in fermentable fiber (notably pectin), essential oils, polyphenols, and flavonoids like hesperidin and naringin [30,31]. These bioactive compounds have been shown to possess antimicrobial, antioxidant, and methane-suppressing properties. For instance, limonene-rich citrus essential oils can inhibit methanogens and protozoa, thereby reducing CH_4_ production while modulating fermentation towards more energetically favorable pathways, such as propionate formation [32,33]. Tannins and other polyphenols present in citrus by-products may further contribute to this effect by disrupting the symbiotic relationship between methanogens and protozoa or by directly inhibiting archaeal activity [34,35]. A potential path of manipulation of rumen methanogenic population could be attributed to the tannin content of the secondary feeds, as well as to an undocumented property of orange as a potent inhibitor of the enzyme hydroxyl methyl glutaryl coenzyme A (HMG-CoA) reductase. As archaea possess unique glycerol-containing membrane lipids linked to long-chain isoprenoid alcohols, which are essential for cell membrane stability, the synthesis of these units in methanogenic archaea is catalyzed by the HMG-CoA reductase. When introducing an inhibitor in the system, the synthesis of isoprenoid units is suppressed, the membrane becomes unstable and cells eventually die, thus reducing methanogen abundance [36]. However, it should be noted, that dose-dependent toxicity is a consistent issue, with moderate tannin levels sometimes exhibiting beneficial properties but high levels causing clear negative physiological outcomes, such as reduced feed intake, decreased nutrient digestibility, and tissue damage [37,38,39].

From a nutritional standpoint, citrus pulp and orange peel silage have been successfully included in sheep and cattle diets, replacing a portion of conventional cereal grains without compromising performance [40,41]. These by-products offer a slowly degradable fiber source that supports rumen function and may reduce the risk of acidosis compared to starch-rich concentrates. Several studies demonstrate that orange waste and citrus by-products can influence rumen bacterial diversity and selectively modulate key taxa. Citrus pectin and orange essential oil supplementation in ruminant diets have showed notable effects in mitigating methane production ranging from transient decreases to sustained reductions up to 19% [42,43,44]. Furthermore, orange waste and citrus by-products like citrus pomace have been found to stimulate growth of fiber-degrading and propionate-producing bacteria such as Butyrivibrio, Fibrobacter, and Prevotella, while reducing methanogenic archaea [44,45,46,47]. Alterations in the fermentation profile have been noted when introducing orange essential oil in heifer nutrition and dried citrus pulp in vitro, in the form of consistent increases in propionate and butyrate analogies, often accompanied by stable or increased ruminal pH [42,43,48]. The identification of taxa like Rikenellaceae RC9 group and Prevotella as responsive to citrus substrates suggests targeted microbial shifts that may underpin improved fermentation and methane mitigation [46,47]. Additionally, microbial fermentation treatments (i.e., with *Saccharomyces cerevisiae*) further enhance protein content and digestibility [31]. Economically, the integration of citrus by-products into ruminant feeding aligns with goals of feed cost reduction and value creation from waste, as they are often available at low or negative cost (when disposal fees are considered), making them attractive alternatives to traditional feed grains [36]. Indeed, local agro-industrial by-products are frequently less expensive on a nutrient basis than primary feed commodities [41,42], and their integration into ruminant rations represents a cost-effective strategy that enhances feed efficiency while reducing the environmental footprint of both the livestock and citrus industries [49,50,51,52]. It should be highlighted though that dosage optimization and safety monitoring for contaminants such as pesticides in citrus by-products are necessary to avoid negatively impacting dry matter intake or nutrient digestibility [42,44,45,53]. Additionally, the transient effects of citrus extract-based additives on methane mitigation observed in some studies imply that continuous supplementation or combination with other mitigation strategies may be required to achieve consistent long-term benefits under commercial settings [48,54].

The present study further investigates the effects of specific dietary components on microbial community composition and activity, emphasizing recent advances in nutritional interventions. In particular, it was designed to test the hypothesis that the dietary inclusion of orange peel waste, either in its processed or unprocessed form, can beneficially modulate the rumen microbial community in dairy sheep, leading to a reduction in methanogenic archaea and associated methane emissions. Specifically, it aims to elucidate how citrus-derived secondary feed ingredients influence microbial composition and fermentation dynamics within the rumen. In addition, the study assesses the comparative efficacy of distinct Next-Generation Sequencing (NGS) primer sets in detecting and characterizing archaeal taxa, with a particular focus on Methanobacteria, thereby addressing methodological biases that may affect microbial community profiling.

## 2. Materials and Methods

### 2.1. Ethical Statement

Experimental procedures were carried out in accordance with the approved protocol by the Research Ethics Committee of the Hellenic Agricultural Organization-DIMITRA regarding animal welfare and handling of animals during experimentations (Protocol no. 429/9 June 2023).

### 2.2. Production of Experimental Feedstuff

A novel valorisation approach was devised to transform orange juice industry by-products into high-value secondary animal feedstuff. The core of the strategy involved enzymatic saccharification of orange peels in a pilot-scale bioreactor, followed by aerobic fermentation of the resulting sugar-rich liquid to cultivate Saccharomyces cerevisiae yeast as a source of single-cell protein. The process of orange peel fermentation and animal feedstuff production has been described in detail by [29]. The saccharification process was optimised using factorial design, with parameters including enzyme dosage (Pectinex and CellicCTec3 at 25 μL/g TS each), solid loading (7.5%), temperature (50 °C), and duration (24 h). The aerobic fermentation of the hydrolysate was also optimised with respect to nutrient supplementation, yeast-to-glucose ratio (0.02), and pH control, conducted at 30 °C for 24 h. Following fermentation, the hydrolysed peels and harvested yeast were stabilised in a pilot drying plant. Both processed (hydrolysed) and unprocessed orange peels were simultaneously dehydrated and milled into a homogeneous coarse powder using a demo-scale rotary drum dryer with a biomass burner, operated at 120 °C for 15–20 h per cycle to ensure microbial stability and feed safety [29].

Prior to the onset of the trial, the basal ration feedstuffs and the mineral/vitamin premix were acquired. In addition, four batches of processed and unprocessed orange peel feed were delivered at the experimental facilities, weighing a total of 250 kg, which were then analyzed as per their chemical composition, determined using the Weende proximate system according to AOAC (2005) procedures [55], while fiber fractions (NDF, ADF, ADL) were analyzed following the Van Soest et al. (1991) detergent system [56] (Table 1).

### 2.3. Animal Trial

The trial took place at the intensive dairy sheep farm of the Research Institute of Animal Science of the Hellenic Agricultural Organization-DIMITRA, in Paralimni, northern Greece. A total of 49 dairy ewes of the Greek indigenous breed of Chios in their 2nd and 3rd lactation after weaning (on day 46 after lambing) were examined for any health issues by a designated veterinarian. Upon their declaration as healthy, and prior to the onset of the study, after they were equally distributed relative to age, body weight and lactation number and cumulative milk yield, a list of 18 animals was produced. The minimum number of ewes was determined so as to ensure the statistical power and validity of the results obtained, without exceeding the number necessary, in accordance with the principles of the 3Rs (“reduction, refinement, and replacement”), taking under consideration the process of rumen fluid collection to be implemented. Prior to the onset of the trial, a power analysis was performed using the General Linear Multivariate Model with Repeated Measures procedure in the GLIMMPSE platform (https://glimmpse.samplesizeshop.org/, RRID: SCR_016297; accessed on 26 March 2023) [57]. The desired power was set at 0.80, with a type I error rate of 0.05. Assuming a moderate effect size (f = 0.40) for treatment, a within-animal correlation of 0.70 across the three sampling points, and three groups with equal sample sizes (*n* = 6 per group), the analysis indicated that this design achieved an estimated power of 0.84. Therefore, the use of six animals per group was considered sufficient to ensure statistical robustness while fully complying with ethical standards to minimize animal use. These animals were allocated randomly to three dietary groups (six animals per group), while ensuring that all groups were homogeneous in terms of average milk yield, mean lactation number, age and body weight. The three groups were designated as Control (C; *n* = 6; lactation number = 2.833 ± 0.389; milk yield = 1833.33 ± 356.965 g/day; age = 3.03 ± 0.323 years and body weight = 61.19 ± 0.781 kg), Processed (Processed; *n* = 6; lactation number = 2.833 ± 0.389; milk yield = 1833.33 ± 418.511 g/day; age = 3.02 ± 0.439 years and body weight = 61.28 ± 0.575 kg), and Unprocessed (Unprocessed; *n* = 6; lactation number = 2.833 ± 0.389; milk yield = 1829.16 ± 352.561 g/day; age = 3.14 ± 0.352 years and body weight = 61.11 ± 0.632 kg), where Processed corresponds to the group receiving ration with the inclusion of dried, processed orange peel feed and Unprocessed corresponds to the group receiving ration with the inclusion of dried, unprocessed orange peel feed. The mean lactation, milk yield, age and body weight of the groups at the start of the trial can be seen in Table 2.

A total of 18 individual feeding stations were constructed to accommodate the respective selected animals. Each feeding station has a total surface of 1.8 m^2^ to ensure the comfort of the animals. The three feeding groups were housed in separate floor pens with a total surface of 25 m^2^ each, and one (float valve) plastic water trough per pen (one per six ewes) with free access was allocated, as well as a feeding trough within each feeding station adapted to the height of the ewes, so that they could feed without any strain on their legs and neck. Special care was taken during the installation of the water troughs, so that they were accessible from all three sides, thus avoiding any obstruction from a singular ewe towards others. The floor pens were under an open-sided barn which had windows on the leeward side, so that wind currents could be formed above the animals and adequate ventilation could be guaranteed, without posing any danger towards the ewes. The floor bedding was wheat straw, refreshed at weekly intervals.

Based on the chemical composition of the orange peel feeds, isonitrogenous and isoenergetic diets were formulated by substituting conventional feed ingredients and by meeting the nutrient requirements of the animals. Diets were formulated to meet nutrient requirements of sheep for lactation [58]. The ingredients and chemical composition of the two experimental diets and the one of the control group can be found in Table 3.

The 18 selected ewes entered the feeding trial on the day after weaning (day 46 postpartum) and remained until the 16th week of lactation, for a total duration of 84 days, with an initial adaptation period during days 1 to 7. The three groups were machine milked, twice daily at 08:00 and 18:00, in the following order: Control, Processed and Unprocessed. Each ewe was offered 1.6 kg of the experimental ration that corresponded to its group, on a daily basis. The concentrate feed was offered right after the morning milking, after being weighted for each ewe, in their individual feeding stations. Once entering the feeding station, the ewes were confined by obstructing the station exit with a wooden barrier, thereby preventing their disengagement, and remained there for 1.5 h until the consumption of the concentrate was completed.

The animals were observed throughout this process on an individual basis and when an ewe completed its meal it was immediately released. Following the previous process, 1.3 kg of alfalfa hay and 0.3 kg of straw were also offered to each ewe daily. Half of the quantity of the roughage was offered after the concentrates in the morning and the rest after the evening milking. Roughage nutrient composition is presented in Table 4.

### 2.4. Sampling and NGS Analysis

Rumen liquid samples from each animal were collected at the beginning and at the end of the feeding trial (days 0 and 84), i.e., 18 samples from each time-point (36 samples in total). The rumen digesta were collected by a veterinarian in the morning following the methodology described by [59,60] before feeding, using an esophageal tube (flexible PVG tube of 1.5 mm thickness and 10 mm I.D.) and a vacuum pump (Selekt Pump, Nimrod, Gloucestershire, UK). The tube had a 30 cm long movable hard plastic cover on the outside to prevent the tube from being worn during its entry into and stay in the ewe’s oral cavity. A plastic spout was also attached to the end of the tube to allow it to penetrate the upper layer of the contents of the rumen. The stomach tube was advanced to a depth of approximately 120 cm and was moved during the collection to ensure an unbiased representative sample. The first 20 mL of rumen digesta was discarded to avoid saliva contamination and, immediately after the collection, liquid samples were stored at −80 °C until their analysis. Finally, using 1 mL of liquid as starting material, microbial DNA was isolated from all 36 rumen digesta samples with the DNeasy PowerSoil Pro Kit (Qiagen, Hilden, Germany). To check the quantity and quality of DNA samples, a Q3000 microvolume spectrophotometer (Quawell, San Jose, CA, USA) was used.

Two different primer pairs that target hypervariable regions of the prokaryotic 16S rRNA gene were used, to assess rumen microbial diversity. For the amplification of the V1–V9 region of the 16S rRNA gene (~1500 base pairs), the following universal bacterial primer pair was selected: S-D-Bact-0008-c-S-20 (5′-AGRGTTYGATYMTGGCTCAG-3′) and 1492R (5′-CGGYTACCTTGTTACGACTT-3′) [61,62]. The second set of primers, Pro341F (5′-CCTACGGGNBGCASCAG-3′) and Pro805R (5′-GACTACNVGGGTATCTAATCC-3′), is a universal prokaryotic primer pair that targets the V3–V4 region of the 16S ribosomal RNA gene and has been used for the simultaneous amplification of bacteria and archaea [63,64,65,66]. All PCRs were performed in 15 µL volumes containing 7.5 µL of the KAPA2G™ Robust HotStart ReadyMix PCR Kit (Kapa Biosystems, Wilmington, MA, USA), PCR-grade water, the primer pair (0.5 µM each) and DNA (~100 ng). For the first primer set, which amplifies the whole 16S gene, cycling conditions included an initial denaturation at 95 °C for 3 min, followed by 35 cycles of 95 °C for 15 s, 55 °C for 30 s and 72 °C for 45 s, with a final extension at 72 °C for 10 min. Regarding the second pair of primers, a touchdown PCR protocol was applied: initial denaturation at 98 °C for 2 min, followed by 35 cycles of 98 °C for 30 s, annealing from 65 °C to 55 °C for 15 s and extension at 68 °C for 30 s, with a final extension at 68 °C for 5 min. The annealing temperature decreased by 1 °C per cycle until reaching 55 °C, which was maintained for the remaining 25 cycles. Finally, all PCR products were subjected to agarose gel electrophoresis to verify successful amplification, while their quality and quantity were also checked with spectrophotometry (Q3000 spectrophotometer).

In order to sequence the 16S amplicons with ligation methodology, these were purified with AMPure^®^ XP beads (Beckman Coulter, Brea, CA, USA) and a DNA library was constructed with the Native Barcoding Kit 96 V14 (SQK-NBD114.96, Oxford Nanopore Technologies plc., Oxford Science Park, Oxford, UK), comprising of 72 pooled and bar-coded PCR amplicons (36 from each primer set). The library was subsequently loaded onto a MinION-Mk1B sequencing platform using a R.10.4.1 flowcell (Oxford Nanopore Technologies plc., Oxford Science Park, Oxford, UK). The obtained raw sequencing data were basecalled and demultiplexed (according to the used barcodes) with Dorado software (Oxford Nanopore Technologies plc., Science Park, Oxford, UK). Additionally, to obtain clean 16S rRNA sequences, these were trimmed with Dorado by removing barcodes, adapter and primer sequences. Finally, reads were filtered based on length (1000–2000 bp for the first primer pair and 350–550 bp for the second primer set) and quality score (QC > 15) criteria.

For taxonomic classification of bacterial and archaeal populations, the bioinformatics workflow ‘wf-16S’ through the open-source platform EPI2ME (Oxford Nanopore Technologies plc., https://epi2me.nanoporetech.com/wfindex/ (accessed on 5 September 2024)) and the database SILVA 138 SSU Ref non-redundant (NR) (https://www.arb-silva.de/documentation/release-123 (accessed on 5 September 2024)) were used, setting 95% as a similarity threshold for successful assignment. For each primer pair, the 36 sequenced PCR products were pooled (one group per sextuplicate) according to feeding trial (groups Control, Processed and Unprocessed) and sampling days (0 and 84) information. In total, six sample groups were created, namely: Control_0, Processed_0, Unprocessed_0, Control_84, Processed_84 and Unprocessed_84. Visualisation of the microbial community composition and abundance in each sample group was achieved by constructing separate taxa bar plots for each primer set with the aforementioned ‘wf-16S’ workflow.

### 2.5. Statistical Analysis

Results were tested for normality using the Shapiro–Wilk test. To evaluate the effects of diet (Processed, Unprocessed, Control) and sampling time (0 h vs. 84 h) on the abundance of Methanobacteria, a Linear Mixed Model (LMM) was applied in R v4.4.3 [67] using the lme4 package [68]. The model included diet, time, and their interaction as fixed effects, with individual animal as a random effect to account for repeated measures across time within each diet group:

Y_ijk_ = μ + D_i_ + T_j_ + (D × T)_ij_ + A_k_ + e_ijk_

where Y_ijk_ denotes the dependent variable (percentages of Methanobacteria); μ the overall mean; D_i_ the fixed effect of the feeding treatment (Processed, Unprocessed and Control) (i = 1–3); T_j_ the fixed effect of the sampling time (Day 0 and Day 84) (j-1-2); (D × T)_ij_ the interaction effects of feeding treatment and sampling time; Ak the random effects of the animal within feeding treatment and e_ijk_ the residual error associated with observation ijk. Statistical significance was considered at *p* < 0.05.

Furthermore, to explore potential associations between Methanobacteria and other microbial groups, we performed Pearson’s correlation analyses within each dietary group (Control, Processed, Unprocessed).

## 3. Results

**Figure 1:**
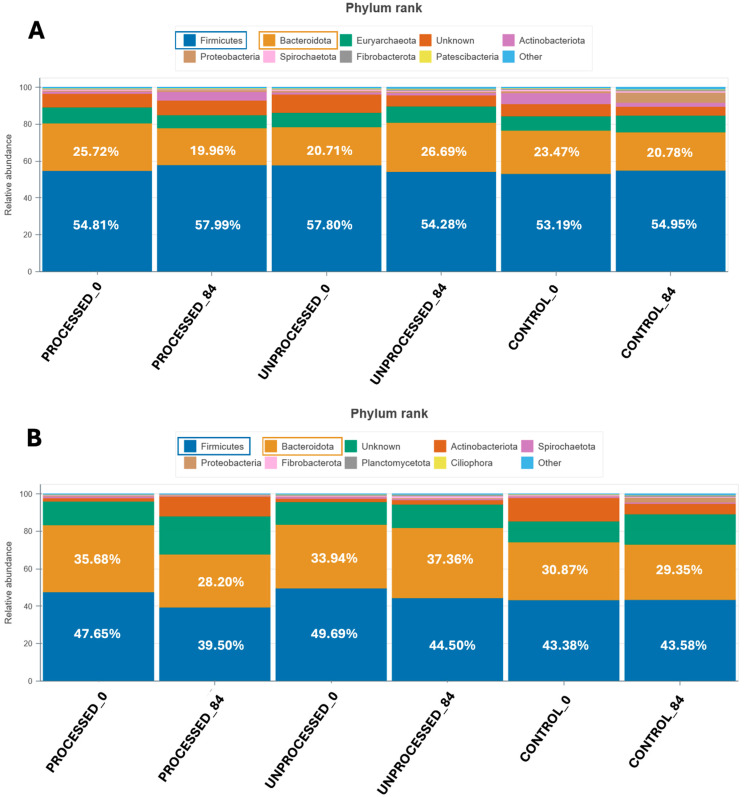
Bar plots showing the relative abundance of microbial phyla, using the prokaryotic primer pair (**A**) and the full 16S primer pair (**B**).

Based on the bar plots representing microbial relative abundances at the phylum level using the two primer pairs (full 16S and prokaryotic), we observed consistent trends across the three treatment groups (processed, unprocessed, and control) over 0 and 84 days of the feeding trial. When comparing the phylum-level profiles from both primer sets, the dominant bacterial phyla were Firmicutes and Bacteroidota across all groups. However, the prokaryotic primer pair (Figure 1A) detected a higher relative abundance of Firmicutes (ranging from 53.19% to 57.99%) and lower Bacteroidota (19.96–26.69%) compared to the full 16S primer set (Figure 1B), where Firmicutes ranged from 39.50% to 49.69% and Bacteroidota showed relatively higher proportions (28.20–37.36%). These discrepancies suggest primer-specific biases in taxonomic recovery, emphasizing the need for adjusting primer selection according to the targeted microbial community.

Beyond primer-related differences at the phylum level, we further examined genus-level interactions between Methanobacteria and fibrolytic bacteria. In the Control group, Methanobacteria was significantly and negatively correlated with Prevotella and Ruminococcus (*p* < 0.05), whereas in the Processed group it showed significant positive associations with Succiniclasticum and Selenomonas (*p* < 0.05), and a negative association with Prevotella (*p* < 0.05). By contrast, no significant correlations were detected in the Unprocessed group (Supplementary Figure S1). In addition to the dominant phyla, both primer sets detected similar secondary phyla, but with some differences in ranking and composition. Specifically, using the prokaryotic primer pair, Euryarchaeota (Archaea), Unknown and Actinobacteriota were the next most abundant groups following Firmicutes and Bacteroidota; while using the full 16S primer pair, the most abundant minor phyla were Unknown, Actinobacteriota and Spirochaetota (Figure 1; see also Supplementary Tables S1 and S2).

Dietary inclusion of orange peel waste also influenced microbiota composition. In both primer sets, the processed and unprocessed inclusions induced shifts in the relative abundances of dominant phyla over the 84-day feeding period. For instance, a modest increase in Bacteroidota and a reduction in Firmicutes were observed in the groups including unprocessed orange peel waste over time, being consistent across both primer pairs. Furthermore, an increase in Proteobacteria was observed at day 84 in the control group, verified also by both primer sets. This increase was not detected in any of the diets containing orange peel waste, suggesting a potential stabilizing or protective effect of citrus-derived components against blooms of Proteobacteria. However, none of these changes were statistically significant (*p* > 0.05) (Supplementary Tables S1 and S2).

**Figure 2:**
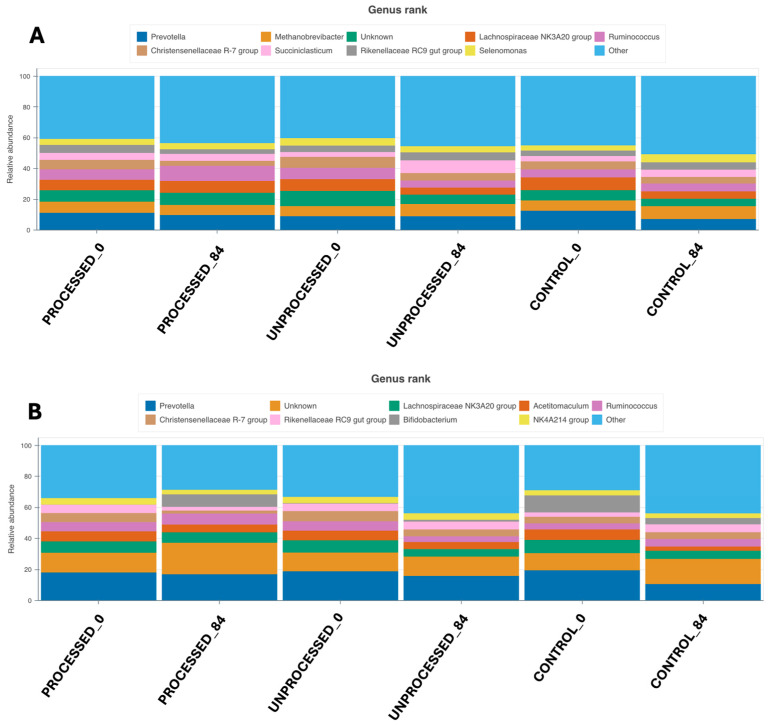
Bar plots showing the relative abundance of microbial genera, using the prokaryotic primer pair (**A**) and the full 16S primer pair (**B**).

While a relatively consistent pattern was observed at higher taxonomic levels (i.e., phylum), greater differences emerged as analysis proceeded to lower taxonomic ranks. Using the prokaryotic primer pair, the most abundant genera included *Prevotella*, *Methanobrevibacter*, *Unknown*, *Lachnospiraceae* and *Ruminococcus*, while in the full-length 16S primer set *Prevotella*, *Unknown*, *Lachnospiraceae*, *Acetitomaculum* and *Ruminococcus* were identified as the most dominant genera. Among the top nine genera, *Succiniclasticum* and *Selenomonas* were exclusively detected with the prokaryotic primer pair, whereas *Bifidobacterium* and *NK4A214* group were identified only with the full-length 16S primer pair. These differences highlight how primer selection influences taxonomic resolution and may lead to the detection or even omission of key microbial groups, especially at finer taxonomic scales (Figure 2; see also Supplementary Tables S3 and S4).

*Prevotella*, being the most dominant genus across both primer sets, exhibited a relatively stable abundance in the diets that were supplemented with processed or unprocessed orange peel waste, with only minor reductions observed at day 84 compared to day 0 (*p* > 0.05). In contrast, a more pronounced decline in *Prevotella* abundance was evident in the control group between 0 and 84 days. This pattern, consistent across both primer pairs, suggests that orange peel by-products may prevent or attenuate the decline of *Prevotella* over time, potentially contributing to the maintenance of microbial functions associated with this genus (Figure 2; see also Supplementary Tables S3 and S4).

**Figure 3:**
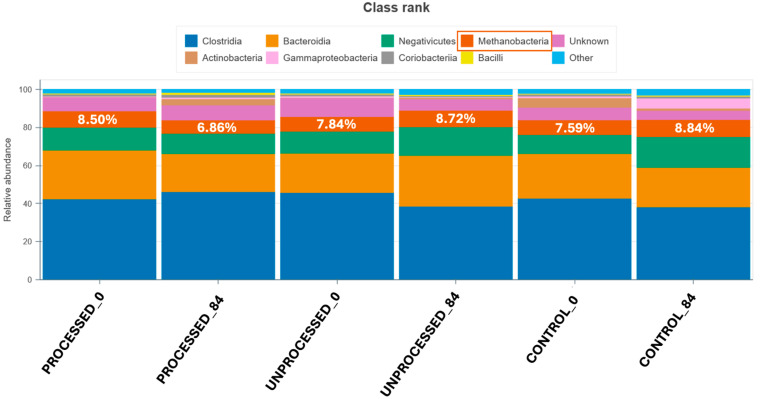
Relative abundance of microbial classes in the six sample groups under study, using the prokaryotic primer pair.

A notable distinction between the two primer pairs used herein was the exclusive detection of Methanobacteria using the prokaryotic primer pair, where it comprised up to 8.84% of the total microbial community. More specifically, it ranged from 6.86% in the Processed_84 group to 8.84% in the Control_84 group. These results indicate that this primer set effectively captured archaeal taxa. In contrast, Methanobacteria were entirely absent when the full-length 16S primer pair was used, suggesting a limited detection capacity for archaeal lineages using the specific primer set (Figure 3).

Interestingly, Methanobacteria abundance increased from day 0 to day 84 in two out of three dietary groups, being the control and unprocessed groups. However, in the group fed with processed orange peel waste, a decrease in Methanobacteria abundance was observed on day 84 compared to day 0 (Figure 3). This selective reduction may indicate that processing of citrus orange peel waste alters its bioactivity, which in turn might lead to reduced methanobacterial abundance. To better illustrate the time-dependent changes in Methanobacteria abundance, relative changes between day-0 and day-84 were also expressed as log_2_ fold change for each group (Processed, Unprocessed, Control). These results are provided in Supplementary Figure S1. Overall, the Processed group showed mostly negative log_2_ fold change values (indicating a decrease at day-84), while the Unprocessed and Control groups displayed more heterogeneous patterns.

**Figure 4:**
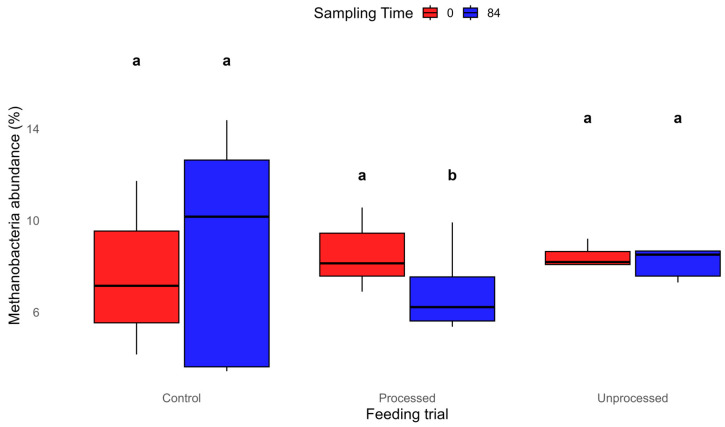
Linear Mixed Model analysis for Methanobacteria abundance (%) by treatment (Control, Processed, Unprocessed) and sampling time (0, 84), superscripts a, b differ at *p* < 0.05.

To complement the descriptive figure, statistical differences related to diet (D), time (T), and their interaction (D × T) were formally tested using a Linear Mixed Model (LMM). The analysis showed no significant overall effect of time (*p* > 0.05) or of the diet × time interaction (*p* > 0.05). However, pairwise contrasts revealed a significant decrease in Methanobacteria abundance in the Processed group between day 0 and day 84 (−19.3%, *p* < 0.05), while no significant changes were observed in Control (*p* > 0.05) or Unprocessed groups (*p* > 0.05) (Figure 4 and Table S5).

Finally, we further examined interactions between Methanobacteria and major fibro-lytic bacterial genera. In the Control group, Methanobacteria was significantly and negatively correlated with Prevotella and Ruminococcus (*p* < 0.05), whereas in the Processed group it showed significant positive associations with Succiniclasticum and Selenomonas (*p* < 0.05), and a negative association with Prevotella (*p* < 0.05). By contrast, no significant correlations were detected in the Unprocessed group (Supplementary Figure S2).

## 4. Discussion

The present study demonstrated that the inclusion of citrus by-products, namely processed and unprocessed orange peel waste, modulated the ruminal microbiota composition—particularly methanogenic populations—throughout an 84-day feeding period. Moreover, the findings highlight the substantial impact of primer pair selection on the accurate detection of archaeal taxa and the characterization of microbial community shifts at finer taxonomic resolutions.

Across both primer sets, Firmicutes and Bacteroidota were identified as the dominant phyla, in agreement with prior studies on ruminant gastrointestinal microbiota [69]. However, the relative abundance of these groups varied depending on the primer set used. The prokaryotic primer pair consistently resulted in a higher Firmicutes level and a lower detection of Bacteroidota, while the full-length 16S primer set resulted in lower differences between these two phyla. The aforementioned differences likely reflect amplification biases inherent to primer design, highlighting the importance of selecting primers that align with the research objective, whether broad community profiling or targeted detection of specific microbial clades [70].

The nutritional manipulation also exerted a measurable effect on microbial composition. Notably, supplementation with orange peel waste, regardless of processing, appeared to attenuate the bloom of Proteobacteria observed in the control group by day 84 (compared to day 0). Given that elevated Proteobacteria levels are frequently associated with microbial dysbiosis and inflammatory conditions [71], this suggests a potentially beneficial role of citrus-derived bioactive compounds in promoting rumen microbial stability.

At lower taxonomic levels, especially the genus level, primer-specific discrepancies became more pronounced. While both primer sets identified *Prevotella*, *Lachnospiraceae* and *Ruminococcus* among the most abundant genera, differences emerged in the detection of other key taxa. For example, *Succiniclasticum* and *Selenomonas* were exclusively observed with the prokaryotic primer set, whereas *Bifidobacterium* and the *NK4A214* group were only detected using the full-length 16S primer pair. These observations reaffirm that primer selection can significantly impact the detection of microbial subpopulations, particularly those of lower abundance but potentially high functional relevance. Despite this, *Prevotella* consistently emerged as the dominant genus across all conditions and primer sets. Interestingly, its abundance remained stable or declined only modestly in the experimental groups (processed and unprocessed), while a greater reduction was observed in the control group. This suggests that orange peel waste supplementation may contribute to the preservation of *Prevotella*-associated functions, such as polysaccharide degradation and short-chain fatty acid (SCFA) production, over time.

One of the most striking findings was the differential detection of Methanobacteria, being captured only by the prokaryotic primer set. The absence of these taxa in the full-length 16S results dataset shows a significant limitation of that primer pair in detecting archaeal lineages, despite their established role in the rumen ecosystem, particularly in methanogenesis [72]. Furthermore, while Methanobacteria abundance increased from day 0 to day 84 in both the control and unprocessed groups, a decrease was observed in the processed group being also statistically significant (*p* < 0.05). This may indicate that thermal or mechanical processing of citrus waste alters its chemical composition, possibly increasing the availability of inhibitory compounds such as limonene (not quantified in this study) that can suppress methanogenic archaea [71].

Importantly, the antimicrobial and antioxidant properties of citrus essential oils offer additional functional benefits. Limonene, the primary compound in orange peel oil, has been shown to modulate rumen microbial activity including suppression of methanogens and protozoa [33]. These microbial shifts hold significance in the context of enteric methane mitigation, which is a critical objective in ruminant production due to methane’s global warming potential and the associated energy losses [3,72]. Several studies have shown the promising potential of dietary supplementation with bitter orange extract (Citrus aurantium/Seville orange) in mitigating enteric methane (CH_4_) emissions, across various cattle production systems. More specifically, significant CH_4_ reductions have been demonstrated in dairy cows [73], beef steers [74], Holstein veal calves [75] and sheep [76] following the inclusion of the extract, alone or in combination with garlic granules (*Allium sativum*). The bioactive compounds present in bitter orange are believed to modulate ruminal fermentation patterns, inhibit methanogenic archaea and promote propionate production.

The present findings align strongly with the increasing evidence that support the inclusion of citrus by-products and citrus-based phytogenics in ruminant diets, as a sustainable and nutritionally viable strategy [77]. In a previous study, inclusion of citrus extract (*Citrus aurantium*) in mid-lactation dairy cows decreased the relative abundance of methanogens, improved rumen fermentation and had a beneficial effect on milk yield [78]. In addition, the nutritional efficacy of citrus by-products, particularly orange peels and citrus pulp, has been well-documented. These by-products offer fermentable fiber, especially pectin and essential bioactive compounds such as polyphenols and limonene-rich essential oils. Their slow degradation rates in the rumen may improve overall fiber digestibility compared to high-starch grains [49], potentially enhancing feed efficiency. Trials involving citrus fruit inclusion in sheep diets demonstrated no negative impact on animal performance parameters, including milk yield, product quality or growth rates [39,40,77,79].

Moreover, the environmental benefits of citrus waste inclusion extend beyond feed substitution. Life-cycle assessments indicate a substantial reduction in CO_2_-equivalent emissions (i.e., up to 0.5 tonnes per tonne of citrus waste repurposed [50,52]. By offsetting the environmental costs of both feed production and waste disposal, the integration of citrus by-products embodies the principles of circular agriculture. Dietary strategies targeting dominant methanogens, especially *Methanobrevibacter* spp., have been shown to reduce methane output while maintaining rumen function. Supplementation with plant secondary metabolites such as tannins and resveratrol has elicited reductions in methane emissions without compromising animal health or performance [30,35]. Citrus by-products, by virtue of their polyphenolic content, may play a similar role, although dose optimization is critical to avoid possible suppression of fiber digestibility observed at higher inclusion levels [80].

The valorization of citrus by-products as alternative feed ingredients presents promising long-term implications for the dairy sheep industry, offering an integrated approach to environmental sustainability, circular bioeconomy, and feed cost reduction. The present findings suggest that processed orange peel waste can contribute to mitigating enteric methane emissions while supporting stable rumen microbiota, thereby enhancing both ecological performance and animal productivity. Such outcomes align with the evolving priorities of low-emission ruminant systems and could facilitate the transition toward carbon-efficient dairy production chains. In the long term, widespread adoption of citrus-derived secondary feedstuffs could lessen the industry’s dependence on imported concentrates, promote regional self-sufficiency, and stimulate synergies between livestock farming and agro-industrial sectors. Nevertheless, this study was limited by its relatively small sample size and the short experimental duration, which may constrain the generalizability of microbiome-level findings to broader production settings. Additionally, while the use of two primer sets provided valuable insights into methodological biases in microbial detection, sequencing depth and bioinformatics pipelines could further influence archaeal resolution and functional interpretation. Future research should therefore aim to integrate metagenomic and metabolomic analyses to elucidate microbial functionality, extend trials under commercial conditions to assess performance and emission outcomes at scale, and explore optimal inclusion rates that balance methane mitigation with nutrient digestibility. It should be highlighted that phytochemicals are widely regarded as affecting rumen fermentation and microbial communities, and the types and amounts of these compounds present in citrus by-products must be thoroughly determined. In doing so, accurate relationships between dietary ingredients and their resulting effects on microbial activity and metabolic processes can be determined. An integrated evaluation combining animal physiology, life-cycle assessment, and techno-economic modelling would further clarify the role of citrus by-products in sustainable dairy sheep nutrition systems.

## 5. Conclusions

Overall, our findings demonstrate that both dietary strategies and methodological approaches (primer selection) can critically influence observed microbiota dynamics. While both processed and unprocessed orange peel waste exerted modulatory effects on microbial community structure, the extent and specificity of these effects varied between taxa and were further shaped by detection methodology. In summary, integrating orange by-products, turned into high-value secondary feedstuffs, in sheep diets offers multidimensional benefits, including methanobacteria reduction. As global livestock systems contend with mounting economic and environmental pressures, alternative feed resources, such as orange peel and other citrus by-products, emerge as dual-purpose solutions that contribute to feed cost reduction and waste valorization while promoting environmentally responsible practices. This supports the feasibility of partially replacing costly conventional concentrates with citrus residues.

## Figures and Tables

**Table 1:** Chemical composition (gr/100gr) of processed and unprocessed orange peel feed used in the feeding trial (dry matter-DM basis except as noted).

Chemical Composition	Processed Orange Peel	Unprocessed Orange Peel
Dry matter (as fed)	94.78	91.27
Crude protein	14.75	7.18
Crude fat	2.96	3.57
Neutral detergent fiber (NDF)	34.00	34.10
Acid detergent fiber (ADF)	22.80	24.80
Lignin Acid Detergent (ADL)	6.40	9.80
Ash	5.84	8.65
Water Soluble Solids	49.00	35.99
Free Glucose	0.67	0.85
Starch	2.51	2.39
Cellulose	11.28	17.47
Hemicellulose	18.40	30.70
Acid Soluble Lignin	0.83	1.06
Total Nitrogen, as a % of the dry matter basis (TN)	9.30	10.70

**Table 2:** Lactation number, milk yield, age and body weight of the three feeding groups at the start of the trial.

Group	Lactation Number	Milk Yield (g/day)	Age (Years)	Body Weight (kg)
Control	2.833 ± 0.389	1833.33 ± 356.965	3.03 ± 0.323	61.19 ± 0.781
Processed	2.833 ± 0.389	1833.33 ± 418.511	3.02 ± 0.439	61.28 ± 0.575
Unprocessed	2.833 ± 0.389	1829.16 ± 352.561	3.14 ± 0.352	61.11 ± 0.632

**Table 3:** Composition of the control and the two experimental diets.

	Diet		
Ingredient Composition (gr/kg of Concentrate as Fed)	Control	Processed Orange Peel	Unprocessed Orange Peel
Corn grain	300	300	300
Barley grain	200	200	200
Wheat grain	200	120	120
Soyabean meal	110	110	110
Sunflower meal	150	120	120
Experimental feedstuff	0	110	110
Limescale	5	5	5
Monocalcium phosphate	5	5	5
Salt	5	5	5
Vitamin and mineral premix	25	25	25
**Chemical composition (g/100 g of DM)**			
Dry matter (DM as fed)	87.59	88.47	88.61
Crude protein	16.94	16.93	15.61
Crude fat	2.18	2.38	2.73
Crude fiber	7.04	6.62	10.35
Neutral detergent fibre (NDF)	17.49	18.36	22.40
Acid detergent fibre (ADF)	8.51	9.31	13.81
Lignin Acid Detergent (ADL)	0.41	1.17	1.53
Ash	2.93	3.17	3.49

**Table 4:** Chemical composition (g/100 g) of alfalfa hay and wheat straw (per dry matter-DM basis except as noted).

Chemical Composition	Alfalfa Hay	Wheat Straw
Dry matter (as fed)	89.21	92.46
Crude protein	20.18	4.78
Crude fat	2.71	1.63
Neutral detergent fibre (om)	60.42	73.12
Acid detergent fibre (om)	32.6	49.41

Om: organic matter.

## Data Availability

The original contributions presented in the study are included in the article, further inquiries can be directed to the corresponding author.

## Supplementary Materials

The following supporting information can be downloaded at: https://www.mdpi.com/article/10.3390/ani15203041/s1, Figure S1: Log_2_ fold change (day 84 vs day 0) in Methanobacteria abundance for each group. Each bar represents an individual paired sample within the Processed, Unprocessed, or Control group. The dashed red line at 0 indicates no change. Negative values correspond to a decrease at day-84 compared with baseline, while positive values indicate an increase. Overall, the Processed group showed mainly negative log_2_ fold change values, whereas the Unprocessed and Control groups exhibited more heterogeneous patterns.; Figure S2: Correlation heatmaps (Processed, Unprocessed, Control). Tiles show Pearson’s r with significance levels: *: *p* ≤ 0.05, ns = not significant. Bold = statistically significant (*p* ≤ 0.05); Table S1: Effects of diet and time on bacterial abundance (%) using the prokaryotic primer pair; Table S2: Effects of diet and time on bacterial abundance (%) using the full 16S primer pair; Table S3: Effects of diet and time on microbial genera abundance (%) using the prokaryotic primer pair; Table S4: Effects of diet and time on microbial genera abundance (%) using the full 16S primer pair; Table S5: Effects of diet and time on Methanobacteria Abundance (%) using the prokaryotic primer pair.

## References

[1] Ponnampalam E.N., Jairath G., Alves S.P., Gadzama I.U., Santhiravel S., Mapiye C., Holman B.W.B. (2025). Sustainable livestock production by utilising forages, supplements, and agricultural by-products: Enhancing productivity, muscle gain, and meat quality—A review. Meat Sci..

[2] Herrero M., Mason-D’Croz D., Thornton P.K., Fanzo J., Rushton J., Godde C., Bellows A.L., de Groot A., Palmer J., Chang J. (2021). Livestock and sustainable food systems: Status, trends, and priority actions. Science and Innovations for Food Systems Transformation.

[3] Gerber P.J., Steinfeld H., Henderson B., Mottet A., Opio C., Dijkman J., Falcucci A., Tempio G. (2013). Tackling Climate Change Through Livestock: A Global Assessment of Emissions and Mitigation Opportunities. https://www.fao.org/4/i3437e/i3437e.pdf.

[4] Patra A.K., Park T., Kim M., Yu Z. (2017). Rumen methanogens and mitigation of methane emission by anti-methanogenic compounds and substances. J. Anim. Sci. Biotechnol..

[5] Hook S.E., Wright A.D.G., McBride B.W. (2010). Methanogens: Methane producers of the rumen and mitigation strategies. Archaea.

[6] Carberry C.A., Waters S.M., Kenny D.A., Creevey C.J. (2014). Rumen methanogenic genotypes differ in abundance according to host residual feed intake phenotype and diet type. Appl. Environ. Microbiol..

[7] Ungerfeld E.M. (2015). Shifts in metabolic hydrogen sinks in the methanogenesis-inhibited ruminal fermentation: A meta-analysis. Front. Microbiol..

[8] Cuervo W., Gómez-López C., DiLorenzo N. (2025). Methane synthesis as a source of energy loss impacting microbial protein synthesis in beef cattle—A review. Methane.

[9] Greening C., Geier R.R., Wang C., Woods L.C., Morales S.E., McDonald M.J., Rushton-Green R., Morgan X.C., Koike S., Leahy S.C. (2018). Alternative hydrogen uptake pathways suppress methane production in ruminants. bioRxiv.

[10] Pereira A.M., Dapkevicius M.d.L.N.E., Borba A.E.S. (2022). Alternative pathways for hydrogen sink originated from the ruminal fermentation of carbohydrates: Which microorganisms are involved in lowering methane emission?. Anim. Microbiome.

[11] Morgavi D.P., Forano E., Martin C., Newbold C.J. (2010). Microbial ecosystem and methanogenesis in ruminants. Animal.

[12] Janssen P.H., Kirs M. (2008). Structure of the archaeal community of the rumen. Appl. Environ. Microbiol..

[13] Danielsson R., Schnürer A., Arthurson V., Bertilsson J. (2012). Methanogenic population and CH_4_ production in Swedish dairy cows fed different levels of forage. Appl. Environ. Microbiol..

[14] Ku-Vera J.C., Jiménez-Ocampo R., Valencia-Salazar S.S., Montoya-Flores M.D., Molina-Botero I.C., Arango J., Gómez-Bravo C.A., Aguilar-Pérez C.F., Solorio-Sánchez F.J. (2020). Role of secondary plant metabolites on enteric methane mitigation in ruminants. Front. Vet. Sci..

[15] Matthews C., Crispie F., Lewis E., Reid M., O’Toole P.W., Cotter P.D. (2019). The rumen microbiome: A crucial consideration when optimising milk and meat production and nitrogen utilisation efficiency. Gut Microbes.

[16] Li X., Norman H.C., Kinley R.D., Laurence M., Wilmot M.G., Bender H., de Nys R., Tomkins N.W. (2016). *Asparagopsis taxiformis* decreases enteric methane production from sheep. Anim. Prod. Sci..

[17] Malyugina S., Holik S., Horký P. (2025). Mitigation strategies for methane emissions in ruminant livestock: A comprehensive review of current approaches and future perspectives. Front. Anim. Sci..

[18] Takahashi L.S., Sanches T., Issakowicz J., Bueno M.S., Bompadre T.F.V., de Paz C.C.P., Abdalla A.L., da Costa R.L.D. (2023). Lipid supplementation with macadamia by-product reduces methane emissions by sheep. Small Rumin. Res..

[19] Niu X., Xing Y., Wang J., Bai L., Xie Y., Sun M., Yang J., Li D. (2024). Effects of *Caragana Korshinskii* tannin on fermentation, methane emission, community of methanogens, and metabolome of rumen in sheep. Front. Microbiol..

[20] Adejoro F.A., Hassen A., Akanmu A.M. (2019). Effect of lipid-encapsulated acacia tannin extract on feed intake, nutrient digestibility and methane emission in sheep. Open Access J..

[21] Muir S.K., Kennedy A.J., Kearney G.A., Hutton P.G., Hutton P.G., Thompson A., Vercoe P., Hill J.O. (2020). Offering subterranean clover can reduce methane emissions compared with perennial ryegrass pastures during late spring and summer in sheep. Anim. Prod. Sci..

[22] Della Rosa M., Sandoval E., Reid P., Luo D., Pacheco D., Janssen P.H., Jonker A. (2022). Substituting ryegrass-based pasture with graded levels of forage rape in the diet of lambs decreases methane emissions and increases propionate, succinate, and primary alcohols in the rumen. J. Anim. Sci..

[23] Sun X., Pacheco D., Luo D. (2016). Forage Brassica: A feed to mitigate enteric methane emissions?. Anim. Prod. Sci..

[24] Ding X.-Z., Long R., Zhang Q., Huang X., Guo X., Mi J. (2012). Reducing methane emissions and the methanogen population in the rumen of tibetan sheep by dietary supplementation with coconut oil. Trop. Anim. Health Prod..

[25] Machmüller A., Soliva C.R., Kreuzer M. (2003). Methane-suppressing effect of myristic acid in sheep as affected by dietary calcium and forage proportion. Br. J. Nutr..

[26] Machmüller A., Dohme F., Soliva C.R., Wanner M., Kreuzer M. (2001). Diet composition affects the level of ruminal methane suppression by medium-chain fatty acids. Crop. Pasture Sci..

[27] Zema D.A., Calabrò P.S., Folino A., Tamburino V., Zappia G., Zimbone S.M. (2018). Valorisation of citrus processing waste: A review. Waste Manag..

[28] de la Torre I., Martin-Dominguez V., Acedos M.G., Esteban J., Santos V.E., Ladero M. (2019). Utilisation/upgrading of orange peel waste from a biological biorefinery perspective. Appl. Microbiol. Biotechnol..

[29] Andrianou C., Passadis K., Malamis D., Moustakas K., Mai S., Barampouti E.M. (2023). Upcycled Animal Feed: Sustainable Solution to Orange Peels Waste. Sustainability.

[30] Alam M.A., Subhan N., Rahman M.M., Uddin S.J., Reza H.M., Sarker S.D. (2014). Effect of citrus flavonoids, naringin and naringenin, on metabolic syndrome and their mechanisms of action. Adv. Nutr..

[31] Oboh G., Ademosun A.O., Lajide L. (2012). Improvement of the nutritive value and antioxidant properties of citrus peels through Saccharomyces cerevisiae solid substrate fermentation for utilization in livestock feed. Livest. Res. Rural. Dev..

[32] Seradj A.R., Abecia L., Crespo J., Villalba D., Fondevila M., Balcells J. (2014). The effect of Bioflavex^®^ and its pure flavonoid components on in vitro fermentation parameters and methane production in rumen fluid from steers given high concentrate diets. Anim. Feed. Sci. Technol..

[33] Al-Sagheer A.A., Abdel Monem U.M., Sayed-Ahmed E.E., Khalil B.A. (2023). Navel orange peel hydroethanolic extract as a phytogenic feed supplement: Impacts on growth, feed intake, nutrient digestibility, and serum metabolites of heat-stressed growing rabbits. Anim. Biotechnol..

[34] Jayanegara A., Leiber F., Kreuzer M. (2012). Meta–analysis of the relationship between dietary tannin level and methane formation in ruminants from in vivo and in vitro experiments. J. Anim. Physiol. Anim. Nutr..

[35] Ngambi J.W., Selapa M.J., Brown D., Manyelo T.G. (2022). The effect of varying levels of purified condensed tannins on performance, blood profile, meat quality and methane emission in male Bapedi sheep fed grass hay and pellet-based diet. Trop. Anim. Health Prod..

[36] Siyal F.A., Wagan R., Bhutto Z.A., Tareen M.H., Arain M.A., Saeed M., Brohi S.A., Soomro R.N. (2016). Effect of orange and banana peels on the growth performance of broilers. Adv. Anim. Vet. Sci..

[37] Besharati M., Maggiolino A., Palangi V., Kaya A., Jabbar M., Eseceli H., De Palo P., Lorenzo J.M. (2022). Tannin in ruminant nutrition. Molecules.

[38] Nawab A., Li G., An L., Nawab Y., Zhao Y., Xiao M., Tang S., Sun C. (2020). The potential effect of dietary tannins on enteric methane emission and ruminant production; as an alternative to antibiotic feed additives—A review. Ann. Anim. Sci..

[39] Singh B., Bhat T.K. (2001). Tannins revisited-changing perceptions of their effects on animal system. Anim. Nutr. Feed. Technol..

[40] Kotsampasi B., Tsiplakou E., Christodoulou C., Mavrommatis A., Mitsiopoulou C., Karaiskou C., Sossidou E., Fragioudakis N., Kapsomenos I., Bampidis V.A. (2018). Effects of dietary orange peel essential oil supplementation on milk yield and composition, and blood and milk antioxidant status of dairy ewes. Anim. Feed. Sci. Technol..

[41] Ferreira A.C.D., Santos A.C.P., de Lima Valença R., Silva B.C.D., Cirne L.G.A., Santana J.C.S., Oliveira V.S., Pereira M.A., Neto J.A.S. (2022). Orange peel silage in lamb feeding improves meat fatty acid profile. Semin. Ciências Agrárias.

[42] Jiménez-Ocampo R., Montoya-Flores M.D., Pámanes-Carrasco G., Herrera-Torres E., Arango J., Estarrón-Espinosa M., Aguilar-Pérez C.F., Araiza-Rosales E.E., Guerrero-Cervantes M., Ku-Vera J.C. (2022). Impact of orange essential oil on enteric methane emissions of heifers fed bermudagrass hay. Front. Vet. Sci..

[43] García-Rodríguez J., Saro C., Mateos I., González J.S., Carro M.D., Ranilla M.J. (2020). Effects of replacing extruded maize by dried citrus pulp in a mixed diet on ruminal fermentation, methane production, and microbial populations in rusitec fermenters. Open Access J..

[44] Geerkens C.H., Schweiggert R.M., Steingass H., Boguhn J., Rodehutscord M., Carle R. (2013). Influence of apple and citrus pectins, processed mango peels, a phenolic mango peel extract, and gallic acid as potential feed supplements on in vitro total gas production and rumen methanogenesis. J. Agric. Food Chem..

[45] Jalal H., Giammarco M., Lanzoni L., Akram M.Z., Mammi L.M.E., Vignola G., Chincarini M., Formigoni A., Fusaro I. (2023). Potential of fruits and vegetable by-products as an alternative feed source for sustainable ruminant nutrition and production: A review. Agriculture.

[46] Yu S., Li L., Zhao H., Tu Y., Liu M., Jiang L., Zhao Y. (2023). Characterization of the dynamic changes of ruminal microbiota colonizing citrus pomace waste during rumen incubation for volatile fatty acid production. Microbiol. Spectr..

[47] Broadway P.R., Callaway T.R., Carroll J.A., Donaldson J.R., Rathmann R.J., Johnson B.J., Cribbs J.T., Durso L.M., Nisbet D.J., Schmidt T.B. (2012). Evaluation of the ruminal bacterial diversity of cattle fed diets containing citrus pulp pellets. Agric. Food Anal. Bacteriol..

[48] Brede J., Peukert M., Egert B., Breves G., Brede M. (2021). Long-Term mootral application impacts methane production and the microbial community in the rumen simulation technique system. Front. Microbiol..

[49] Alnaimy A., Gad A.E., Mustafa M.M., Atta M.A.A., Basuony H.A.M. (2017). Using of citrus by-products in farm animals feeding. Open Access J. Sci..

[50] Negro V., Ruggeri B., Fino D., Tonini D. (2017). Life cycle assessment of orange peel waste management. Resour. Conserv. Recycl..

[51] Khan M.I., Ashraf U., Mubarik U. (2023). Industrial application of orange peel waste: A review. Int. J. Agric. Biosci..

[52] Dilek F.B., Barampouti E.M., Mai S., Moustakas K., Malamis D., Martin D.S., Yetis U. (2025). Orange peel waste valorization: An integrated assessment of environmental and economic sustainability in animal feed production. Waste Biomass Valorization.

[53] Ahmed E., Gaafar A.M., Nishida T. (2024). Agro–industrial by–products as ruminant feed: Nutritive value and in vitro rumen fermentation evaluation. Anim. Sci. J..

[54] Roque B.M., van Lingen H.J., Vrancken H., Kebreab E. (2019). Effect of mootral-a garlic- and citrus-extract-based feed additive-on enteric methane emissions in feedlot cattle. Transl. Anim. Sci..

[55] AOAC (2005). Official Methods of Analysis of AOAC International.

[56] Van Soest P.J., Robertson J.B., Lewis B.A. (1991). Methods for dietary fiber, neutral detergent fiber, and nonstarch polysaccharides in relation to animal nutrition. J. Dairy Sci..

[57] Glimmpse 3.1.3. https://glimmpse.samplesizeshop.org/.

[58] National Research Council (2007). Nutrient Requirements of Small Ruminants: Sheep, Goats, Cervids, and New World Camelids.

[59] Ramos-Morales E., Arco-Pérez A., Martín-García A.I., Yáñez-Ruiz D.R., Frutos P., Hervás G. (2014). Use of stomach tubing as an alternative to rumen cannulation to study ruminal fermentation and microbiota in sheep and goats. Anim. Feed. Sci. Technol..

[60] Mould F.L., Kliem K.E., Morgan R., Mauricio R.M. (2005). In vitro microbial inoculum: A review of its function and properties. Anim. Feed. Sci. Technol..

[61] Klindworth A., Pruesse E., Schweer T., Peplies J., Quast C., Horn M., Glöckner F.O. (2013). Evaluation of general 16S ribosomal RNA gene PCR primers for classical and next-generation sequencing-based diversity studies. Nucleic Acids Res..

[62] Matsuo Y., Komiya S., Yasumizu Y., Yasuoka Y., Mizushima K., Takagi T., Kryukov K., Fukuda A., Morimoto Y., Naito Y. (2021). Full-length 16S rRNA gene amplicon analysis of human gut microbiota using MinION™ nanopore sequencing confers species-level resolution. BMC Microbiol..

[63] Takai K.E.N., Horikoshi K. (2000). Rapid detection and quantification of members of the archaeal community by quantitative PCR using fluorogenic probes. Appl. Environ. Microbiol..

[64] Herlemann D.P., Labrenz M., Jürgens K., Bertilsson S., Waniek J.J., Andersson A.F. (2011). Transitions in bacterial communities along the 2000 km salinity gradient of the Baltic Sea. ISME J..

[65] Takahashi S., Tomita J., Nishioka K., Hisada T., Nishijima M. (2014). Development of a prokaryotic universal primer for simultaneous analysis of bacteria and archaea using next-generation sequencing. PLoS ONE.

[66] Alvanou M.V., Loukovitis D., Melfou K., Giantsis I.A. (2024). Utility of dairy microbiome as a tool for authentication and traceability. Open Life Sci..

[67] R Core Team R: A Language and Environment for Statistical Computing 2024; R Foundation for Statistical Computing, Vienna, Austria. https://www.R-project.org/.

[68] Bates D., Mächler M., Bolker B., Walker S. (2015). Fitting Linear Mixed-Effects Models Using lme4. J. Stat. Softw..

[69] Jami E., Mizrahi I. (2012). Composition and similarity of bovine rumen microbiota across individual animals. PLoS ONE.

[70] Shin N.R., Whon T.W., Bae J.W. (2015). Proteobacteria: Microbial signature of dysbiosis in gut microbiota. Trends Biotechnol..

[71] Camargo F.P., Sakamoto I.K., Duarte I.C.S., Silva E.L., Varesche M.B.A. (2021). Metataxonomic characterization of bacterial and archaeal community involved in hydrogen and methane production from citrus peel waste (*Citrus sinensis* L. Osbeck) in batch reactors. Biomass Bioenergy.

[72] Johnson K.A., Johnson D.E. (1995). Methane emissions from cattle. J. Anim. Sci..

[73] Khurana R., Brand T., Tapio I., Bayat A.R. (2023). Effect of a garlic and citrus extract supplement on performance, rumen fermentation, methane production, and rumen microbiome of dairy cows. J. Dairy Sci..

[74] Bitsie B., Osorio A.M., Henry D.D., Silva B.C., Godoi L.A., Supapong C., Brand T., Schoonmaker J.P. (2022). Enteric methane emissions, growth, and carcass characteristics of feedlot steers fed a garlic-and citrus-based feed additive in diets with three different forage concentrations. J. Anim. Sci..

[75] Brand T., Miller M., Kand D. (2021). Effect of natural feed supplement on methane mitigation potential and performance in Holstein bull calves. Open J. Anim. Sci..

[76] Ahmed E., Batbekh B., Fukuma N., Kand D., Hanada M., Nishida T. (2021). A garlic and citrus extract: Impacts on behavior, feed intake, rumen fermentation, and digestibility in sheep. Anim. Feed. Sci. Technol..

[77] Kasapidou E., Mitlianga P., Basdagianni Z., Papatzimos G., Mai S., Barampouti E.M., Papadopoulos V., Karatzia M.-A. (2025). Orange peel feed ingredient in lactating ewes: Effect on yoghurt chemical composition, fatty acid profile, antioxidant activity, physicochemical properties, and sensory quality. Appl. Sci..

[78] Yu S., Li L., Zhao H., Zhang S., Tu Y., Liu M., Zhao Y., Jiang L. (2023). Dietary citrus flavonoid extract improves lactational performance through modulating rumen microbiome and metabolites in dairy cows. Food Funct..

[79] Bampidis V.A., Robinson P.H. (2006). Citrus by-products as ruminant feeds: A review. Anim. Feed. Sci. Technol..

[80] Nam I.S., Garnsworthy P.C., Ahn J.H. (2006). Supplementation of essential oil extracted from citrus peel to animal feeds decreases microbial activity and aflatoxin contamination without disrupting in vitro ruminal fermentation. Asian-Australas. J. Anim. Sci..

