# Supplementary material for: Primer-Dependent Insights into Rumen Microbiota and Methanogen Shifts Induced by Orange Peel Secondary Feed in Dairy Sheep
Source: Animals (Basel). 2025 Oct 20;15(20):3041. doi: 10.3390/ani15203041 (PMC12561016; doi:10.3390/ani15203041)
Supplement: Supplementary file 1 [file animals-15-03041-s001.zip › animals-3874571-supplementary.pdf]

Primer-Dependent Insights into Rumen Microbiota and Methanogen Shifts Induced by Orange Peel Waste Secondary Feed in Dairy Sheep Rations

Supplementary materials

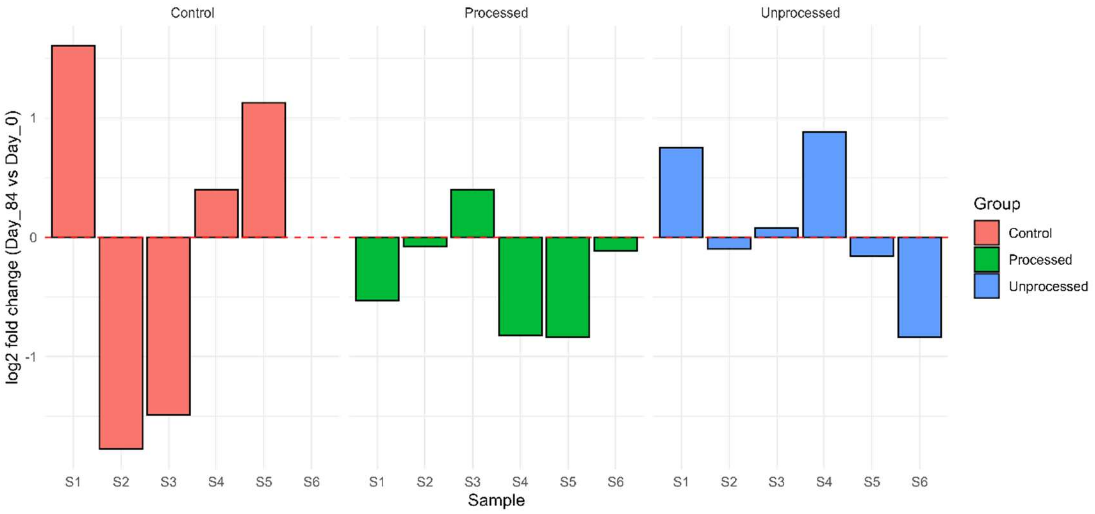

Figure S1. Log_2 fold change (day 84 vs day 0) in Methanobacteria abundance for each group. Each bar represents an individual paired sample within the Processed, Unprocessed, or Control group. The dashed red line at 0 indicates no change. Negative values correspond to a decrease at day-84 compared with baseline, while positive values indicate an increase. Overall, the Processed group showed mainly negative log_2 fold change values, whereas the Unprocessed and Control groups exhibited more heterogeneous patterns.

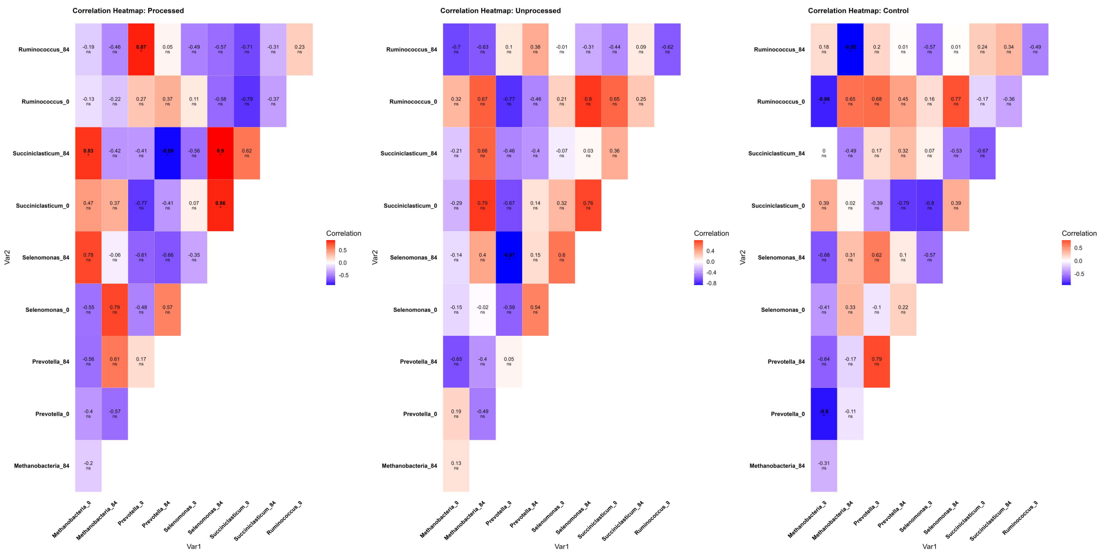

Figure S2 Correlation heatmaps (Processed, Unprocessed, Control). Tiles show Pearson's r with significance levels: *: p ≤ 0.05, ns = not significant. Bold = statistically significant (p ≤ 0.05).

Table S1 Effects of diet and time on bacterial abundance (%) using the prokaryotic primer pair

	**Feeding Trial**				**Time**			**Significance**		
Variable	Control n=12	Processed n=12	Unprocessed n=12	SED_D	Day 0 n=18	Day 84 n=18	SED_T	D	T	DxT
Firmicutes	53.88	55.92	55.66	2.063	54.81	55.50	1.669	NS	NS	NS
Bacteroidota	22.31	23.14	23.91	2.099	23.60	22.64	1.698	NS	NS	NS
Proteobacteria	3.15	0.73	0.76	1.540	0.78	2.32	1.246	NS	NS	NS

SED_D = standard error of difference for diet; SED_T = standard error of difference for time. NS: not significant. Results are based on analyses performed using the prokaryotic primer pair.

Table S2 Effects of diet and time on bacterial abundance (%) using the full 16S primer pair

	**Feeding Trial**				**Time**			**Significance**		
Variable	Control n=12	Processed n=12	Unprocessed n=12	SED_D	Day 0 n=18	Day 84 n=18	SED_T	D	T	Dx T
Firmicutes	43	43.59	46.83	2.496	47.06	41.9	2.02	NS	NS	NS
Bacteroidota	30.49	31.57	35.67	1.906	33.61	31.54	1.542	NS	NS	NS
Proteobacteria	1.51	0.55	0.33	0.828	0.33	1.26	0.67	NS	NS	NS

SED_D = standard error of difference for diet; SED_T = standard error of difference for time. NS: not significant. Results are based on analyses performed using the full 16S primer pair.

Table S3 Effects of diet and time on microbial genera abundance (%) using the prokaryotic primer pair

	Feeding Trial				Time			Significance		
Variable	Control n=12	Processed n=12	Unprocessed n=12	SED_D	Day 0 n=18	Day 84 n=18	SED_T	D	T	DxT
Prevotella	10.41	11.06	9.35	2.377	11.4	9.15	1.923	NS	NS	NS
Selenomonas	4.48	4.04	4.6	1.073	4.27	4.47	0.571	NS	NS	NS
Succiniclasticum	4.09	4.6	5.84	1.31	3.68	6.01	1.06	NS	*	NS
Ruminococcus	5.1	8.16	5.98	0.819	6.43	6.4	0.663	**	NS	**

SED_D = standard error of difference for diet; SED_T = standard error of difference for time. NS: not significant; *: p < 0.05; ** : p < 0.01. Results are based on analyses performed using the prokaryotic primer.

Table S4 Effects of diet and time on microbial genera abundance (%) using the full 16S primer pair

	Feeding Trial				Time			Significance		
Variable	Control n=12	Processed n=12	Unprocessed n=12	SED_D	Day 0 n=18	Day 84 n=18	SED_T	D	T	DxT
Prevotella	15.14	16.88	17.61	2.78	18.9	14.18	2.222	NS	NS	NS
Ruminococcus	4.4	6.76	4.95	0.728	5.39	5.34	0.589	**	NS	NS

SED_D = standard error of difference for diet; SED_T = standard error of difference for time. NS: not significant; ** : p < 0.01. Results are based on analyses performed using the full 16S primer pair.

Table S5 Effects of diet and time on Methanobacteria Abundance (%) using the prokaryotic primer pair

	Feeding Trial				Time			Significance		
Variable	Control n=12	Processed n=12	Unprocessed n=12	SED_D	Day 0 n=18	Day 84 n=18	SED_T	D	T	DxT
Methanobacteria Abundance (%)	8.219	7.680	8.278	0.843	7.976	8.142	0.684	NS	**	NS
Significance (LMM contrasts)	NS (0 vs 84)	p < 0.05 (–19.3%)	NS (0 vs 84)							

SED_D = standard error of difference for diet; SED_T = standard error of difference for time. NS: not significant; ** : p < 0.01. Significance (LMM contrasts) refers to pairwise comparisons of Day 0 vs Day 84 within each diet. Results are based on analyses performed using the prokaryotic primer pair.
